# Evaluating the effectiveness of psychosocial resilience training for heart health, and the added value of promoting physical activity: a cluster randomized trial of the *READY *program

**DOI:** 10.1186/1471-2458-9-427

**Published:** 2009-11-23

**Authors:** Nicola W Burton, Kenneth I Pakenham, Wendy J Brown

**Affiliations:** 1 The University of Queensland School of Human Movement Studies, Brisbane, Australia; 2 The University of Queensland School of Psychology, Brisbane, Australia

## Abstract

**Background:**

Depression and poor social support are significant risk factors for coronary heart disease (CHD), and stress and anxiety can trigger coronary events. People experiencing such psychosocial difficulties are more likely to be physically inactive, which is also an independent risk factor for CHD. Resilience training can target these risk factors, but there is little research evaluating the effectiveness of such programs. This paper describes the design and measures of a study to evaluate a resilience training program (*READY*) to promote psychosocial well-being for heart health, and the added value of integrating physical activity promotion.

**Methods/Design:**

In a cluster randomized trial, 95 participants will be allocated to either a waitlist or one of two intervention conditions. Both intervention conditions will receive a 10 × 2.5 hour group resilience training program (*READY*) over 13 weeks. The program targets five protective factors identified from empirical evidence and analyzed as mediating variables: positive emotions, cognitive flexibility, social support, life meaning, and active coping. Resilience enhancement strategies reflect the six core Acceptance and Commitment Therapy processes (values, mindfulness, defusion, acceptance, self-as-context, committed action) and Cognitive Behavior Therapy strategies such as relaxation training and social support building skills. Sessions include psychoeducation, discussions, experiential exercises, and home assignments. One intervention condition will include an additional session and ongoing content promoting physical activity. Measurement will occur at baseline, two weeks post intervention, and at eight weeks follow-up, and will include questionnaires, pedometer step logs, and physical and hematological measures. Primary outcome measures will include self-reported indicators of psychosocial well-being and depression. Secondary outcome measures will include self-reported indicators of stress, anxiety and physical activity, and objective indicators of CHD risk (blood glucose, cholesterol [mmol·L^-1^], triglycerides, blood pressure). Process measures of attendance, engagement and fidelity will also be conducted. Linear analyses will be used to examine group differences in the outcome measures, and the product of coefficients method will be used to examine mediated effects.

**Discussion:**

If successful, this program will provide an innovative means by which to promote psychosocial well-being for heart health in the general population. The program could also be adapted to promote well-being in other at risk population subgroups.

**Trial registration:**

ACTRN12608000017325.

## Background

### Psychosocial Risks for Coronary Heart Disease

There is strong and consistent evidence of a causal association between both depression and social isolation or lack of quality social support, and the causes and prognosis of coronary heart disease (CHD) [1]. Acute life-event stress can trigger coronary events [1,2], and anxiety can be involved in the onset of CHD [3]. Poor psychosocial functioning may impact on cardiovascular health via biological mechanisms such as cortisol hypersecretion and disturbed hypothalamic-pituitary-adrenal axis functioning, hypertension and blood pressure reactivity to stress, impaired endothelial and vascular functioning, pro-inflammatory cytokines, platelet aggregation and activation and impaired fibrinolysis, atherogenic lipid profiles, and the development of metabolic syndrome [2,4].

### Resilience Training to Improve Psychosocial Well-being and Promote Heart Health

Identifying effective interventions to reduce depression, stress and anxiety, and improve social connectedness is therefore, a potential means of preventing and managing CHD. Resilience training targets modifiable intra-personal skills and protective factors so as to increase an individual's hardiness for remaining psychologically and physically healthy (i.e., resilient) in the face of cumulative stress. Five key psychosocial protective factors are (a) positive emotions, (b) cognitive flexibility, (c) life meaning, (d) social support, and (e) active coping strategies [5]. Each of these factors has been related to lower levels of depressive symptomatology, lower risk of CHD, and better health outcomes for those already diagnosed with CHD [5,6].

Most resilience studies explore the personal characteristics associated with effective coping or resilience, and focus on young people or individuals experiencing specific adverse circumstances (e.g., illness, bereavement, abuse). There are very few controlled studies investigating resilience training with adults. A worksite trial of a 5 × 8 hour program, implemented over five weeks, demonstrated significantly higher levels of self esteem, locus of control, purpose in life, and interpersonal relations among program participants compared with a control group [7]. A trial of an adaptation of this program with 10 × 90 minute modules, implemented over five weeks, for people with diabetes found no significant difference between program participants and a usual care group on measures of the same psychosocial outcomes or physiological health, although there was some suggestion of improved diabetes-related coping [8]. Participants of a worksite trial of an (approximate) 14 day program, implemented over six months, for employees with illnesses attributed to work stress demonstrated higher levels of effective coping (including seeking social support) and lower levels of depression compared with baseline [9]. While there are many studies examining stress management as part of cardiac rehabilitation [10], we are not aware of any studies that have used resilience training to promote heart health.

### Integrating Physical Activity Promotion with Psychosocial Resilience Training for Heart Health

People experiencing psychosocial difficulties, such as depression, social isolation and life stress, are also likely to be physically inactive [11,12], which is an independent risk factor for CHD [13,14]. Epidemiological evidence suggests at least a 30% median risk reduction in cardiovascular disease for those doing regular activity [15], and it has been estimated that for every 1% increase in population levels of physical activity, up to 100 deaths from CHD could be avoided [16]. Physical activity can also reduce cardiovascular risk factors such as hypertension, hyperlipidaemia, and obesity [15]. Physical activity promotion is therefore, an important component of heart health programs.

Physical activity has also been identified as a potential coping resource [5] that can provide enduring resilience to stress [17]. There are numerous studies indicating a positive association between physical activity and psychosocial well-being [18-20]. Physical activity can protect against incident depression symptoms [21-23], and meta-analyses of treatment studies indicate large effect sizes (-0.8, -1.3) for depression symptoms [20,24]. This evidence provides a justification for integrating physical activity promotion with interventions promoting psychosocial well-being.

Authors of other resilience studies have discussed a possible positive association between resilience training and physical activity. Qualitative feedback from the Waite et al., program indicated that some program participants commenced a fitness regime, even though exercise was not directly covered by the training [7]. Although Bradshaw et al., found no significant group difference for self reported exercise, program participants were significantly less likely than those in the control group to report barriers to physical activity, or trouble exercising because of not liking exercise or health problems [8]. It is not clear however, to what extent physical activity was specifically promoted during this program, although it is likely to have been identified as part of diabetes management. The added value of promoting physical activity as part of resilience training is therefore unclear.

### Aim

We have developed a psychosocial resilience training program (*READY*: REsilience and Activity for every DaY) that promotes psychosocial well-being in adults. The aim of this paper is to describe the design and measures of a study to investigate whether the program can improve psychosocial functioning and lower CHD risk indicators, and the added value of integrating physical activity promotion.

## Methods/Design

### Study Design

**Figure 1:**
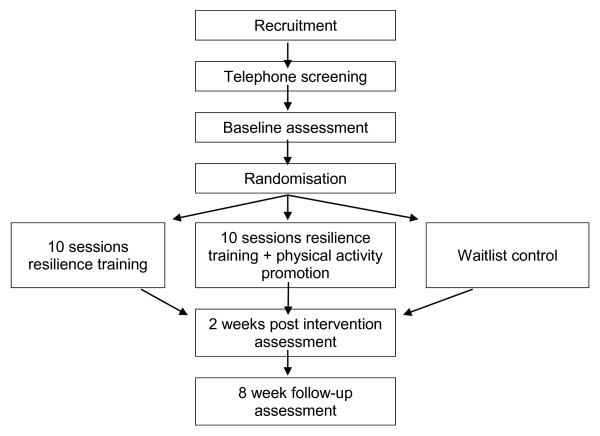
**Overview of study process**.

Figure 1 provides an overview of the study process. This is a cluster randomized trial with two intervention conditions and a wait list group. Participants in one intervention condition will receive the psychosocial resilience training program (*READY*). Participants in the other intervention condition will also receive the resilience training program, as well as additional content promoting physical activity. Measurements will take place at baseline, two weeks post intervention and after an additional eight weeks, and will include questionnaires, pedometer step logs, physical measures and hematological data. The study protocol was approved by The University of Queensland Medical Research Ethics Committee (2007000303).

### Setting

The study will take place in Townsville, Australia. Townsville is a regional city in the state of Queensland with a population of approximately 175,550 people. Compared with the state of Queensland, the median age of residents (33 years) is younger, the median household income is slightly higher, and the unemployment rate is comparable [25].

### Participants and Randomization

Participants will be volunteers recruited from staff of the Townsville district of the State Government Department of Education and Training. The study will be advertised using information sessions and email lists, and potential participants will submit a written expression of interest. Respondents will be initially screened over the telephone by a research assistant to exclude those who (a) are currently receiving psychiatric/psychological treatment (counseling or pharmacological) or who have previously been diagnosed with a major psychiatric disorder (e.g. schizophrenia), or (b) have a medical condition which contraindicates physical activity participation as suggested by the 2005 Sports Medicine Australia screening guidelines [26].

Eligible respondents will be provided with study details, and those who provide verbal consent will be scheduled for baseline assessment. Participants attending baseline assessment will receive written and verbal information on the study requirements and asked to provide written consent before any data are collected. After baseline assessment, participants will be clustered by occupation (teachers/teacher aides, management/senior staff, administration/support staff) and geographical location (Townsville City, Regional). Each cluster will be randomly allocated to one of the three study conditions by a research assistant simultaneously drawing clusters and conditions out of two separate containers.

### Sample Size

The sample size is based on detecting a difference in personal well being as measured by Ryff's Scales of Psychological Well-Being [27]. Previous research showed that differences of 3.0 points (SD 4.0) can be detected post intervention [28]. To achieve a similar difference, using a two tailed test with alpha level 0.05 and power of 80%, we will require 28 participants in each of the three groups. Allowing for attrition, we will aim to recruit 30-35 participants per condition.

### Blinding

It is not possible for the participants or interventionists to be blinded to the intervention conditions, as individuals will be aware of the program information delivered. Participants will be advised that the study involves the same resilience program, with one group receiving a new session module. A research officer will conduct the measurement sessions, and may become aware of a participant's group allocation during their interaction with participants. The principal investigator (NB) will be responsible for data analyses.

### Compliance and Loss to Follow-up

Except for those who explicitly request no further involvement in the study, all participants will be asked to complete the baseline, post-intervention and follow-up assessment. Attendance at intervention sessions will be recorded, and individuals who miss two sequential sessions (unplanned) will be contacted by phone and encouraged to return. Data will be analyzed on an intention to treat basis.

### Intervention

#### Group 1: READY resilience program

The *READY *program targets five key psychosocial protective factors of (a) positive emotions, (b) cognitive flexibility, (c) life meaning, (d) social support, and (e) active coping strategies that were identified from empirical literature [5]. The intervention approach is based on Acceptance and Commitment Therapy (ACT) [29]. ACT is an empirically based third generation Cognitive Behavioral Therapy that uses acceptance and mindfulness strategies, and commitment and behavior change strategies to produce psychological flexibility and resilience through six core processes: acceptance, cognitive defusion (changing our relationship with thoughts), being present (mindfulness), self-as-context, values and committed action [29]. Reviews of ACT randomized control trials show an overall advantage of ACT compared with control conditions [29-31].

An outline of the *READY *program sessions is presented in Table 1. Each session includes psychoeducation on the protective factor(s) targeted, group discussion, individual reflection, experiential exercises and opportunities to learn practical strategies. Home assignments build on information presented in the sessions and include reflection, self-monitoring and skills practice. Participants receive a detailed workbook that includes program notes (highlighting key themes), sections for critical reflection, learning activities to complete, and inspirational quotations. The learning and reflection activities comprise the *READY *personal plan, which is an individualized resource for participants to apply the generalized information to their specific context and personal style.

**Table 1:** Overview of *READY *Resilience Training Sessions

Session Topic	Protective Factor Targeted
Introduction and Program Overview *READY *resilience model, protective factors, warning signs of low resilience.	
Activity Promotion (group 2 only) Activity benefits, national activity guidelines, step counting & pedometers, identifying preferences, goal setting, barriers & problem solving.	Physical activity
Mindfulness Mindfulness vs mindlessness, mindfulness strategies.	Cognitive flexibility Positive emotions
Defusion 1 Thought fusion, identifying unhelpful thoughts, defusion strategies.	Cognitive flexibility
Defusion 2 Observer self, defusion strategies.	Cognitive flexibility
Acceptance About emotions, responding to emotions, about acceptance, acceptance strategies.	Positive emotions
Mid Program Review Resilient and non resilient characteristics, activating and trouble shooting coping strategies.	Active coping
Life Values and Meaningful Action About values, value identification, meaningful action.	Life meaning
Social Connectedness Types of support, support responses, active and reflective listening, barriers to social connectedness.	Social support
Relaxation and Pleasant Activities Active relaxation exercises, progressive muscle relaxation exercises, pleasurable activities.	Positive emotions
Review and Planning for the Future Resilient and non resilient characteristics, activating and trouble shooting coping strategies.	Active coping

Note. Although different themes are identified for each session, the content in each session is referred to and followed up in subsequent sessions, so as to integrate the material across sessions.

The program will be implemented as 10 × 2.5 hour sessions over 13 weeks (one school term and two weeks of school holidays). Sessions will be in a group format with 10-20 participants per group, and will be led by an experienced psychologist. A detailed trainer's manual has been prepared including learning objectives, outlines, learning activities, handouts and audiovisual materials for each session. Interventionists will be trained by the program authors (NB and KP).

#### Group 2: READY resilience program plus physical activity promotion

Participants will receive the resilience training described for Group 1, as well as an additional session and ongoing content promoting physical activity. The session will include psycho-education on physical activity and resilience; a review of national physical activity guidelines; discussion of step counting and pedometer use; consideration of preferred types of physical activity; goal setting; strategies to redress barriers to physical activity, and planning and problem solving for relapsing participation. Walking, both purposive and incidental, will be specifically promoted as a physical activity option requiring no resources. Participants will be given a pedometer (step counter) and logbook, and will receive instruction and rehearsal on how to wear and use the pedometer for self-monitoring. Subsequent sessions will include a check on progress with physical activity goals, and discussion on how to apply resilience strategies to facilitate physical activity participation e.g., obtaining social support, identifying associated values, using cognitive strategies to redress negative thoughts.

### Wait list Condition

Participants allocated to this condition will receive no contact during the study period, apart from measurement sessions. Participants will be offered the most efficacious intervention condition after the trial.

### Measures

Participants will attend group assessment sessions at a local venue. Data will be collected at baseline, two weeks after the intervention period, and again after a further eight weeks. Self-report data will include questionnaire information on psychosocial functioning, physical health, physical activity participation, sociodemographic characteristics (measured only at baseline), and program feedback (measured at post-intervention and follow-up). Participants will be asked to provide pedometer step counts for seven consecutive days. Physical data will include objectively measured height and weight, waist circumference and systolic and diastolic blood pressure. Haematological data will require a finger prick capillary blood sample to assess triglycerides, cholesterol and glucose. Process measures will include participant attendance and self-rated involvement with home assignments, as well as assessment of the fidelity of the intervention delivery.

#### Primary outcome measures

A self-administered battery of psychological questionnaires will be used to derive outcome measures. Unless stated, all items use the previous three months as the referent period.

1. Ryff's Scales of Psychological Well-Being assesses six areas: autonomy, environmental mastery, personal growth, positive relations with others, purpose in life, and self-acceptance [27]. Each sub-scale consists of nine items and responses are scored on a six point Likert scale (1 *strongly disagree *to 6 *strongly agree*). This measure is widely used, has well established reliability and validity [6], and has previously been shown to be sensitive to detecting treatment effects [28].

2. The Center for Epidemiological Studies Depression Scale (CES-D) has 20 items and assesses self reported symptoms associated with depression [32], including affective, somatic, cognitive, motivational and interpersonal dimensions. Responses are scored on a four point Likert scale (0 *rarely or none of the time [less than one day/week] *to 3 *most or all of the time [5-7 days/week]*). This is one of the most commonly used self report questionnaires on depression for the general (vs clinical) population, and has established reliability and validity [32].

#### Secondary outcome measures

The following measures will also be used. Sequentially, resting blood pressure will take place prior to the finger-prick blood sample and then the physical measures. Questionnaires will be done during the assessment session.

1. The Short Version of the Depression Anxiety Stress Scale (DASS-21) has 21 items and consists of three subscales: depression, anxiety and stress [33]. Items ask respondents to indicate the frequency of intra-personal experiences, such as finding it hard to wind down, on a four point Likert scale (0 *not at all *to 3 *most of the time*). Each subscale has demonstrated high internal consistency and yielded meaningful discriminations in a variety of settings in both Australian clinical and community samples [33].

2. Physical activity participation will be assessed via self-report using items adapted from the Active Australia surveys to assess the total time spent during the last week (i) walking briskly for recreation or exercise (adapted to exclude walking to get to and from place to place), (ii) doing moderate physical activity, like social tennis, swimming, golf, (iii) doing vigorous physical activity like aerobics, cycling, running [34]. Two additional items will be used to assess total time spent walking for transport and cycling for transport. Total time spent in recreational physical activity will be calculated by summing time spent in minutes across the three categories, with time in vigorous activity weighted by a factor of two to reflect greater intensity i.e., Σ[minutes walking + minutes moderate activity + (minutes vigorous activity × 2)]. Total time spent in transport activity will be calculated by summing time spent in minutes of walking and cycling for transport. Physical activity will also be assessed using pedometer step counts. Participants will be asked to wear a pedometer for seven consecutive days and record the total number of steps taken each day; this data will be used to derive average daily step counts.

3. Physical indicators of CHD risk. Resting blood pressure will be assessed via an aneroid sphygmomanometer (Alpk2 Aneroid sphygmomanometer, Model No. 500-V, Japan), with two measures taken at least one-minute apart, and the average of the readings used in the analyses [35]. If the variation between the two measures exceeds 5%, a third measure will be taken in order to reduce any potential measurement error. Waist circumference will be measured in duplicate at the narrowest point between the lower rib and the iliac crest at the end of a normal expiration [36] with a flexible steel tape (F10-02, KDS, Japan) by an ISAK-accredited Level 1 Anthropometrist. If the variation between two measures exceeds 5% a third measure will be taken in order to reduce any potential measurement error. Body mass index will be calculated using height measured using a portable stadiometer (Portable Height Scale, Mentone Educational Center, Australia) to the nearest 0.01 m and body weight to the nearest 0.1 kg via calibrated bioelectrical impedance scales (Tanita TBF-521, Tanita Corporation, Tokyo).

4. Hemotological measures will be assessed using a 35 μl capillary blood sample drawn from a finger prick blood sample for immediate analysis via a Cholestech LDX system (Cholestech LDX, Cholestech, USA). Each capillary blood sample will be analyzed for random blood glucose (mmol·L^-1^), total cholesterol (mmol·L^-1^), HDL-cholesterol (mmol·L^-1^), LDL-cholesterol (mmol·L^-1^) and triglycerides (mmol·L^-1^).

5. Somatic complaints. Participants will be asked to indicate on a five point Likert scale (1 *rarely or never *to 5 *almost daily*) how often they were distressed by each of 14 somatic complaints, such as sleep difficulties, muscular tension, headaches and stomach pains.

#### Mediating variables

**Figure 2:**
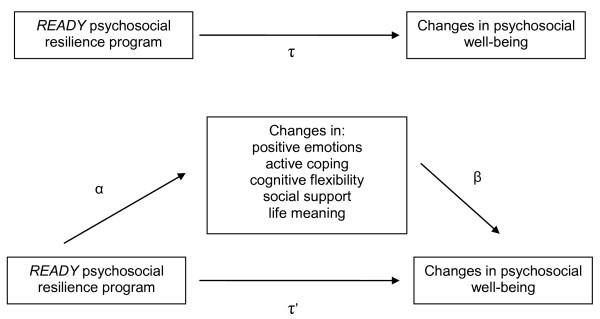
**Conceptual meditational model**.

The *READY *program targets five protective factors that will be evaluated as mediating variables. The conceptual relationship between these variables and outcome measures is presented in Figure 2.

1. Positive emotions. The Positive and Negative Affect Schedule (PANAS-X) is widely used with well established psychometric properties [37]. We will use the ten items positive affect subscale. Respondents indicate on a five point Likert scale (1 *very slightly or not at all *to 5 *extremely*) how often they have felt each of ten feelings (e.g., enthusiastic, interested, determined).

2. Active Coping: The Brief COPE assesses 14 different coping strategies (e.g., humour, active coping and planning, denial and self blame, and behavioral disengagement) [38]. We will use the active coping subscales. Respondents indicate to what extent they use each of the specified strategies to try and cope with stress, using a four point Likert scale (0 *I haven't been doing this at all *to 3 *I've been doing this a lot*). The Brief COPE is comparable with the full COPE, and has acceptable levels of factorial validity and reliability [38].

3. Meaning. The Valued Living Questionnaire [39] will be adapted to exclude items assessing the importance of each life domain. Participants are asked to rate how consistent their actions have been in accordance with their life values for each of ten life domains (e.g., family relationships, community life) using a seven point Likert scale (1 *very inconsistent *to 7 *very consistent*).

4. Cognitive Flexibility. Two questionnaires will be used. The Mindful Attention Awareness Scale has 15 items to assess experiences of mindlessness in daily circumstances such as tasks and social interactions [40] using a six point Likert scale (1 *almost always *to 6 *almost never*. The scale has been shown to be reliable, valid, and sensitive to change [40]. The 10 item Acceptance and Action Questionnaire II assesses psychological flexibility and experiential avoidance using a seven point Likert scale (1 *never true *to 7 *always true*). Psychometric analyses indicate acceptable levels of factorial validity, criterion validity and reliability [29].

5. Social Support. Four items from The Brief COPE [38] will be used to assess use of emotional and instrumental support. Analyses of the Brief COPE [38] and the COPE [38] indicated that these four items loaded on a single factor [38], and had acceptable internal reliability.

#### Sociodemographic characteristics

Questionnaires will be used to assess gender, age, height, weight, educational qualifications, occupation, household composition, and gross annual household income.

#### Process measures

Participants' attendance at each session will be recorded by the interventionist, and the percentage attended of the total number of sessions will be calculated. Participants will be asked each week to rate their level of engagement with the home assignments for that session, using a five point Likert scale (1 *I was unable to do the homework this week *to 5 *I did all the activities*). At post intervention and follow-up, participants will be asked to provide feedback on their overall satisfaction with the program and materials, using a 5 point Likert scale (1 *strongly disagree *to 5 *strongly agree*) and open ended questions. To assess the fidelity of the intervention as delivered, two sessions by each interventionist will be videorecorded and rated by the program authors (NB, KP) for extent of adherence to the trainer's manual.

### Analyses

Data will be analyzed on an intention-to-treat basis, using random effects mixed modeling to examine group differences in the outcome measures among baseline, post-intervention and follow-up. Linear analyses will be used to examine the potential mediation of the five identified protective factors (see Figure 2). We will calculate the (a) direct effect of the program on the outcome measures (τ); (b) effect of the intervention on each of the mediating variables, adjusted for baseline values (α); (c) the effect of the residual change score (post intervention score adjusted for the baseline score) of the proposed mediating variables on the outcome measures, after adjustment for the intervention (β); and then examine whether there is attenuation or change in significance in the relationship between the intervention and the outcome measures, after adjustment for the change in the mediating variables (τ'). The product of the α and β coefficients will be used to provide an estimate of the magnitude of the mediation effect in the units of the outcome variable, and the 95% confidence intervals of the point estimates of the mediated effects will be examined to test for mediation [41].

## Discussion

Little work has been done to evaluate the effectiveness of resilience training with adults in the general population. We are not aware of any studies that have used resilience training to reduce psychosocial and physical indicators of CHD risk, or that have assessed the added value of physical activity promotion. This paper describes the design and methods of a trial of the *READY *psychosocial resilience training program, which will provide data on each of these issues. This study is consistent with positive psychology which focuses on the strengths of individuals that enables them to thrive, and the call for prevention and health promotion research to integrate psychosocial (e.g., depression) and behavioral (e.g., physical activity) risk factors [42].

This study is innovative in a number of ways. Unlike cross sectional studies that assess the concurrence of personal characteristics and resilience, this is an intervention study of resilience *training*. The program targets otherwise healthy adults in the general population rather than children or adults in specific adverse circumstances. This is a novel application of resilience training; to promote heart health by focusing on identified CHD risk factors of depression, poor social support and life stress. Subsequently, the assessment framework incorporates indicators of both psychosocial (well-being, depression) and physical (blood pressure, lipids, blood glucose) health. As it is a protective factor for both heart health and psychosocial well-being, we have integrated physical activity promotion with the resilience program, and will examine the added value of doing so.

At a conceptual level, this study will contribute to our understanding of resilience. The mediational analyses will allow us to evaluate our model of resilience that targets five protective factors (positive emotions, cognitive flexibility, life meaning, active coping, social support) to promote psychosocial well-being (ie., conceptual theory test), and the independent contribution of each of the protective factors in promoting change. We will also be able to evaluate the extent to which the *READY *program impacts on each of these protective factors as hypothesized mechanisms of change (i.e., action theory test).

There are some methodological issues to consider with this study. Recruitment of staff from a regional district of a government department may result in a study sample that is not representative of the general population. As participants will be recruited from within one organization, there may be a risk of contamination between intervention conditions if they communicate with each other. However the clustering of individuals by occupational group and geographical location may reduce this risk. Participants receiving the physical activity promotion will receive one more intervention session than those who receive only the psychosocial resilience training. It is unlikely however, that the additional two and a half hour session, in the context of the 25 hour program, will by itself provide marked additional benefits. To minimize potential trainer effects, interventionists will be involved in both group conditions (i.e. resilience training AND resilience training + physical activity promotion).

## Conclusion

If effective, the *READY *program will demonstrate an innovative means to improve psychosocial well-being and thereby promote heart health in adults in the general population. We will also be able to assess the added value of integrating physical activity promotion and resilience training. If the program is effective, it could be disseminated through similar training programs in worksites or community settings, or adapted to use written or web-based self-help materials. The program could also be used to promote psychosocial well-being in any population subgroups identified as at risk of depression, poor social support and life stress.

## Abbreviations

CHD: Coronary Heart Disease.

## Competing interests

The authors declare that they have no competing interests.

## Authors' contributions

All authors contributed to the conceptualization and design of the study. NB led the overall process, KP led the psychological measurement component, and WB led the physical measurement component. NB and KP developed the *READY *program. NB led the writing for this manuscript with contributions and critical comment from the other two authors. All authors read and approved the manuscript.

## Pre-publication history

The pre-publication history for this paper can be accessed here:

http://www.biomedcentral.com/1471-2458/9/427/prepub
